# Knowns and unknowns about the neurobiology of stuttering

**DOI:** 10.1371/journal.pbio.3002492

**Published:** 2024-02-22

**Authors:** Nicole E. Neef, Soo-Eun Chang

**Affiliations:** 1 Institute for Diagnostic and Interventional Neuroradiology, University Medical Center Göttingen, Göttingen, Germany; 2 Department of Psychiatry, University of Michigan, Ann Arbor, Michigan, United States of America; 3 Department of Communication Disorders, Ewha Womans University, Seoul, Korea

## Abstract

Stuttering occurs in early childhood during a dynamic phase of brain and behavioral development. The latest studies examining children at ages close to this critical developmental period have identified early brain alterations that are most likely linked to stuttering, while spontaneous recovery appears related to increased inter-area connectivity. By contrast, therapy-driven improvement in adults is associated with a functional reorganization within and beyond the speech network. The etiology of stuttering, however, remains enigmatic. This Unsolved Mystery highlights critical questions and points to neuroimaging findings that could inspire future research to uncover how genetics, interacting neural hierarchies, social context, and reward circuitry contribute to the many facets of stuttering.

## Abstract 2

The origin of stuttering is closely linked to early brain development in children but ultimately unknown. This Unsolved Mystery highlights critical questions that could inspire future research to uncover how genetics, interacting neural hierarchies, social context, and reward circuitry contribute to the many facets of stuttering.

## Introduction

You are at your favorite bakery, craving a pain au chocolat. As you attempt to order, your jaw, lips, and tongue will not move and no air flows through your voice box—you struggle to get the speech sound out, but however much you try, you are frozen in place and there is no sound. You cannot answer the clerk’s friendly greeting, and to your dismay you can now see that she looks uncomfortable, unsure of how to react. Looking to escape the awkward situation, you end up ordering something you do not want at all, only because it has a name that you can say without trouble. Such avoidance strategies might help for the moment, but for the person who recounted this experience, such situations have been familiar since childhood, recur continuously, and their speech impediment worsens in crucial social contexts such as during flirting, job interviews, or meetings.

Among communication disorders, stuttering is one of the most frequently occurring, far exceeding laryngeal dystonia, aphasia, and speech issues resulting from Parkinson’s disease, combined [1–6]. It is a speech disorder in which speakers know exactly what they want to say and can coordinate and move their vocal organs, but intermittent loss of control occurs, where articulator movements freeze or fall into an idle loop during instances of stuttering. The overt primary symptoms are speech blocks, sound and syllable repetitions, and sound prolongations that disrupt the usual fluid, automatic process of speech production. Concomitant physical behaviors such as eye-blinking, grimacing, or extraneous limb and body movements can accompany stuttering. The covert consequences range from shame and frustration to social anxiety. Stuttering can severely limit the scope of interpersonal verbal communication and set up barriers that impact education and employment outcomes [7].

Stuttering onset occurs either abruptly or gradually during early childhood between the ages of 2 and 5 [5,6,8]. Up to 8% of preschool-age children begin to stutter and about a fifth retain lifelong stuttering [5]. Around the same developmental period of stuttering onset, children strengthen their articulatory system [9], expand their active vocabulary [10], learn to combine words into longer and more complex phrases [11], and progress in acquiring their language’s typical rhythm and prosody patterns [12]. Besides speech and language development, at that age, children also acquire cognitive, social, and emotional skills to navigate through day-to-day communicative contexts. Thus, in many cases, stuttering emerges while the brain features a remarkable capacity for plasticity. Children’s brain structure and function development is intricately influenced by their experiences, reactions, and interactions. This complexity poses a significant challenge in disentangling the contributions of both genetic predisposition (nature) and environmental influences (nurture) to the occurrence and remission of stuttering.

Clarification of the neurobiological basis of stuttering is made even more difficult by the fluctuation, variability, and heterogeneity of the symptoms. Specifically, stuttering is characterized by intermittency within stuttering persons and variability across the stuttering population. Within-person intermittency is reflected in the variability of symptom severity across days, weeks, and months [13,14]. Moreover, visible and audible features, and thus, overt severity of symptoms, varies with personality, social context, communicative goals, and affective state [15,16]. In addition to overt speech behaviors, persons who stutter can develop a rich spectrum of strategies to hide their stuttering, for example, by avoiding sounds or words, rephrasing utterances, or inserting interjections and starter or filler words. Like overt stuttering, the degree of covertness varies across individuals and situations. How the severity of one’s own stuttering, whether overt or covert, is perceived and experienced also varies from person to person [17]. It must therefore be noted that the intermittency of stuttering, the great heterogeneity of stuttering behavior, and the individual internal experience of stuttering pose challenges for its operationalization. However, the majority of neuroimaging studies conducted thus far have primarily centered on overt stuttering as a key behavioral feature for examining brain correlates [18]. Against this background it becomes clear that our current knowledge about the neurobiological underpinnings of stuttering must be incomplete.

Considering the relative high incidence and significant psychosocial impacts, we still know relatively little about the etiology of stuttering. From a neuroscientific perspective, it is an idiopathic condition with multiple compelling characteristics—breakdown in speech motor control at its core [19,20], with severity of symptoms influenced, but not caused by, higher-order language [21], cognitive [22], or emotional processes [23]. Fundamental questions related to the nature of stuttering remain unanswered. In this Unsolved Mystery, we highlight many such questions, organized under major themes, and research advances relevant to the neurobiology of stuttering that have contributed to our understanding of this complex speech condition.

### Is stuttering genetic?

Genetic research on people who stutter has the potential to not only provide windows into key molecular–biological conditions of the disorder, but also to enhance our knowledge about the neurobiological mechanisms enabling humans to acquire and produce fluent speech. As in most cases of developmental speech and language impairments, stuttering is a rich phenotype and likely involves a complex genetic architecture that results from the inheritance of multiple risk factors with small individual influences [24,25].

The first clues for a genetic contribution were drawn from twin-based heritability studies showing that the probability for both twins to stutter is substantially higher in monozygotic than in dizygotic twins [26]. Heritability was further demonstrated by the high positive rate of family history. Approximately 50% of individuals who stutter report at least 1 additional relative who stutters, as estimated across clinically ascertained cohorts [7]. However, because the heritability is substantially less than 100%, environmental risk factors must also contribute.

The sex bias gives a further hint of a genetic contribution. Stuttering persists in 1% of the adult population, predominantly in males with a male-to-female ratio of 1:4. By contrast, at stuttering onset in early childhood, the male-to-female ratio is 1:2 [5]. As epidemiological data indicate that affected females recover more often from stuttering than affected males, it can be assumed that sex-related neurobiological factors influence the brain’s capacity to recover from stuttering; however, the underlying neurodevelopmental mechanisms are unknown.

Advances in genetic sequencing enabled linkage studies in large consanguineous families with multiple affected members. In such families, stuttering has been repeatedly linked to rare variants of lysosomal targeting pathway genes (*GNPTAB*, *GNPTG*, *NAGPA*, *AP4E1*) [27,28] and further novel chromosomal loci [29,30]. These promising discoveries gave reason for more recent neuroimaging studies to explore a link between risk genes and brain structures [29,31–33]. However, although identifying variants of functional significance in co-segregation patterns of large families helps detect causality, only variation of the same risk loci in multiple unrelated individuals who stutter would establish truly functional links. A first genome-wide association study of developmental stuttering was not able to replicate previously identified loci, controverting the relevance of previously identified genes to the general population with stuttering [34]. The limited success can be explained largely by the small sample size that is insufficient for detecting single-nucleotide polymorphisms with anticipated small effects against a high background of incidental variation of individual genomes [35]. Robust associations may require tens of thousands of genotyped and phenotyped participants [25]. To overcome this significant lack of statistical power, it would be beneficial to extend clinical routines by collecting DNA samples for sequencing and including standardized cloud-based test batteries for documenting the variance in phenotypes of stuttering.

At present, new high-throughput and massively parallel DNA sequencing technologies are requiring substantially reduced costs and time than previous technologies to sequence an entire human genome. Such whole-genome sequencing has already begun to link molecular pathways that regulate gene expression during early brain development to speech and language impairments [36]. Population-level biological and clinical data may help identify clinical–molecular–biochemical subtypes of stuttering in the future.

### What are the neural markers of persistent stuttering?

When using current imaging technologies to examine the brain of an individual who stutters, it is unlikely that we will detect apparent morphological or functional anomalies. Both gray and white matter structures will appear well-formed and in their proper places in persons who stutter, and initial observations of brain waves will not appear atypical. Stuttering does not exhibit overt signs like a visible fracture in an X-ray or the clear disorganized electrical activity seen in an electrocardiogram for cardiac arrhythmia. However, the perspective shifts when comparing data from different groups of individuals. Through the lens of statistical analysis, it becomes possible to identify subtle neural deviations. More than 2 decades of brain research studies have accumulated evidence for structural and functional neural correlates of stuttering.

Both children and adults who stutter show atypical brain structure and functional patterns that can be localized and form part of a number of major neural networks. Implicated are cortical areas of the speech motor planning and control networks (Box 1), including frontal lobe regions such as the motor cortex, premotor cortex, inferior frontal gyrus, frontal operculum, insular cortex, and presupplementary and supplementary motor areas [37–39]. Also implicated are parietal and temporal perisylvian regions, such as the supramarginal gyrus, and higher order auditory regions, which might be related to differences in sensorimotor integration and feedback control [37,38,40]. Furthermore, subcortical structures such as the basal ganglia, thalamus, and cerebellum are implicated, which may relate to differences in learning, initiation, timing, sequencing, and error monitoring functions [37,38,41–45]. Morphological differences in limbic brain regions involved in reward processing and emotion regulation, such as the nucleus accumbens and amygdala are associated with persistent stuttering [46–48]. Along with dysfunctional gray matter regions, alterations have also been reported for white matter structures, including the arcuate fasciculus, superior longitudinal fasciculus, frontal aslant tract, corticobulbar tracts, and cerebellar penduncles, which are responsible for transmitting information between brain regions involved in speech production and motor control [49–52]. Comprehensive reviews of both state and trait markers of stuttering have been conducted and are available in the existing literature [53–59].

**Box 1. The vocal motor system.**

**Fig 1:**
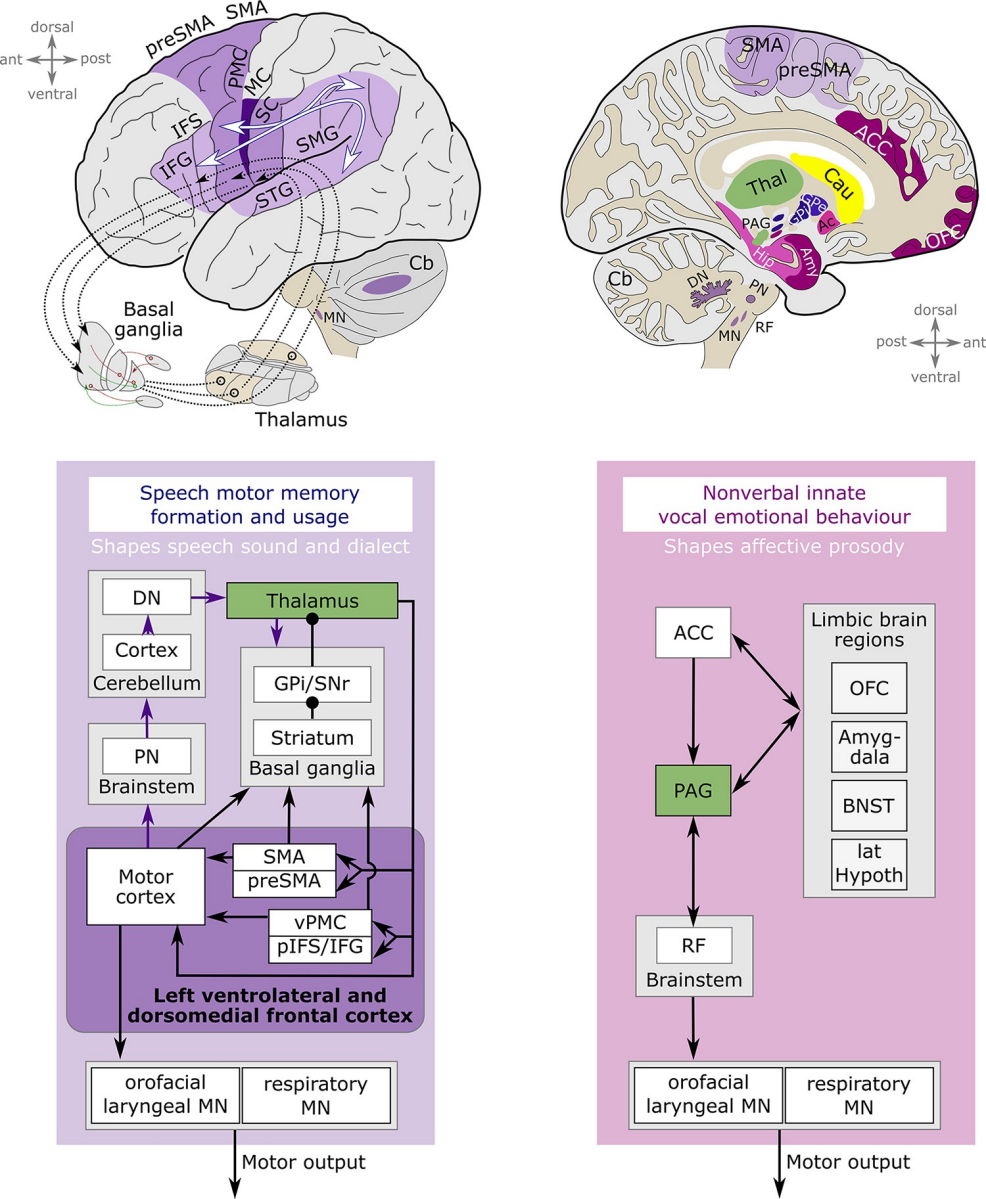
Box 1—The vocal motor system.

Comparative studies between vocalizing vertebrates, including teleost fish, songbirds, and mammals, suggest that hierarchically organized motor pathways contribute to vocal behavior [60–64]. Together with clinical case studies, neuroimaging, and direct electrical stimulation in humans, respective findings mainly shape our current understanding of the neuroanatomy of speech. Transcallosal and cortico–cortical connections between speech motor, auditory, and somatosensory areas, cortico–thalamo–cortical loops via the basal ganglia, and cerebello–thalamo–basal ganglia pathways are believed to have a crucial role in supporting speech learning and automatization [60,65–68], particularly within a critical sensorimotor learning period. The automatization of chunked speech motor sequence output might engage synapses between the premotor and motor cortex [60,65]. Ultimately, within the mature vocal speech system, a set of left ventrolateral and dorsomedial frontal brain areas are key to the volitional initiation of speech [61,69,70], propagating their output towards orofacial and respiratory motor neurons in the brainstem to drive our speech organs (Fig 1).

Besides a volitional articulatory motor network (Fig 1), marked with purple colors in the image, a primary vocal motor network (Fig 1), marked with deep magenta colors in the image, runs from the anterior cingulate cortex via the periaqueductal gray into the reticular formation of pons and medulla oblongata, and from there to the orofacial and laryngeal motoneurons [61,68,71]. The anterior cingulate cortex and the periaqueductal gray receive input from limbic brain structures including the amygdala, bed nucleus of the striata terminalis, and lateral hypothalamus [71,72]. The periaqueductal gray seems to control the readiness to vocalize in nonhuman primates and to gate social and playfulness vocalization in rodents [73,74]. The anterior cingulate cortex is well positioned to integrate cognition, emotion, and action. Its electrical stimulation in humans elicits facial displays, interoceptive sensations, autonomic responses, and laughter and smiling display, indicating that it may orchestrate social emotional behavior [75].

Human speech is based on a considerably broader array of brain regions, and the intricate network of connections within the human connectome is notably more extensive. Depicted brain regions and circuits (Fig 1) constitute core actors in the machinery of vocal speech production, and while all depicted components of the volitional articulatory motor network have been associated with stuttering, components of the primary vocal motor network also seem to be involved, including the anterior cingulate cortex, amygdala, and orbitofrontal cortex.

Abbreviations: Ac, nucleus accumbens; ACC, anterior cingulate cortex; Amy, amygdala; BNST, bed nucleus of the stria terminalis; Cau, caudate nucleus; Cb, cerebellum; DN, dentate nucleus; Gpe, globus pallidus external segment; Gpi, globus pallidus internal segment; Hip, hippocampus; IFG, inferior frontal gyrus; lat Hypoth, lateral hypothalamus; MN, vocal tract motor neurons; pIFS, posterior inferior frontal sulcus; PMC, premotor cortex; MC, primary motor cortex; OFC, orbitofrontal cortex; PAG, periaqueductal gray; PN, pontine nuclei; RF, reticular formation, specifically the lateral reticular formation and the nucleus retroambiguus; SC, primary somatosensory cortex; SMA, supplementary motor area; SMG, supramarginal gyrus; STG, superior temporal gyrus; Thal, Thalamus; vPMC, ventral premotor cortex.

Furthermore, in children with persistent stuttering, MRI techniques have shown morphological differences in cortical and subcortical motor structures, including decreases in cortical thickness in the left premotor and motor regions [76], and decreases in gray matter volume in the left ventral premotor cortex and subcortical areas, including the basal ganglia [77]. White matter structure differences have also been observed, affecting areas involved in auditory–motor integration, motor initiation, monitoring, and interhemispheric coordination [77–80]. These structural differences are correlated with stuttering severity [77], suggesting their relevance to speech fluency.

During spontaneous speech production, children with persistent stuttering exhibit decreased brain activity in the left premotor cortex and basal ganglia compared to children who do not stutter [81]. Additionally, studies indicate initial evidence of large-scale neural network connectivity differences in children with persistent stuttering, particularly involving interactions between speech motor networks and other cognitive control networks [82].

What are the most striking differences between brain abnormalities in adults and children who stutter? In contrast to children, adults frequently exhibit heightened speech-related activity and connectivity within the right hemisphere cortical structures, encompassing frontal and parietal regions, rolandic operculum, and insula [37,38,83]. This pronounced activation in the right hemisphere exceeds that of the corresponding speech-related areas in the left hemisphere [56,84,85], leading to long-standing discussions regarding its potential role as a compensatory mechanism [39,84]. The absence of a similar rightward shift in children [81] supports this proposed compensatory hypothesis. However, task-related MRI data in children are scarce, and recent studies present a more diverse perspective, suggesting that the alterations observed in the right hemisphere may represent a combination of both compensatory efforts and direct manifestations of stuttering [51,80,85].

In contrast to adults, a recent report showed that children who would go on to have persistent stuttering exhibit significantly reduced volume of the putamen early in development [77]. However, this discrepancy diminishes with age, and an intriguing paradox emerges during adulthood, where adults who stutter exhibit increased neural activity within the basal ganglia, including the putamen [86] and caudate nucleus [41]. One plausible hypothesis is that an early-occurring structural variance in the basal ganglia may initially contribute to the observed group difference, but this distinction normalizes over time due to developmental cascades influencing interconnected brain structures. For example, the structural variance in the basal ganglia early in development and its connection to the supplementary motor area may potentially exert an effect on distinct developmental trajectories in regions such as the thalamus and cerebellum. Further investigations are warranted to elucidate the precise mechanisms underlying these developmental dynamics in individuals who stutter.

Altogether, imaging findings suggest that stuttering, like other complex disorders, can be attributed to network-level disruptions [82,87–89]. Implicated networks include core hubs of speech motor skill acquisition and automatization, sensorimotor integration, feedback and error monitoring, cognition and goal-directed behavior, and limbic structures coordinating affect and social context. These networks integrate common processes implicated in verbal communication, involving multiple time scales from milliseconds for voicing features to seconds for prosodic features. Thus, fluent speech requires the neural organization of hierarchical motor sequence representations that are stable enough to be automatically executed and at the same time flexible enough to adapt to affective, social, and goal-directed demands.

Despite the research advances reviewed in this section, answers to the following questions remain elusive. How does the critical period of speech acquisition shape interactions between speech motor learning circuits and speech motor production circuits in typically developing and stuttering children? How and when does the developing system switch between states of learning, states of automatization, and states that require monitoring via the central executive or sensory feedback control systems? What are the underlying neural circuits responsible for transitioning between pure habitual execution of speech movements and states that necessitate implementing prosodic modulations based on social context (e.g., speaking to a pet, friend, or an authority figure) and affective state (e.g., feeling pleased or angry)? How does the brain encode hierarchical speech motor sequence representations? And how is the initiation of speech motor sequences influenced by hierarchical structures or different cognitive and affective states?

### What facilitates spontaneous recovery in children who stutter?

Spontaneous recovery from stuttering is common in children, reported to be 80% or more [5], and neural recovery patterns may give us insights into the neural basis of fluent speech production and its pathologies. Unlike therapy-induced speech fluency learned during adulthood, spontaneous recovery during childhood results in complete alleviation of symptoms, with no effort or internal struggle to produce fluent speech. Though there are no definitive objective markers, several behavioral and demographic factors are associated with childhood recovery from stuttering. These factors include female sex, no family history of stuttering, younger age at stuttering onset, higher scores on speech sound accuracy, higher expressive and receptive language scores, and lower stuttering frequency [90,91]. Other factors, such as performance on a nonword repetition task [92] and time since stuttering onset [90,93], have also been reported.

**Fig 2:**
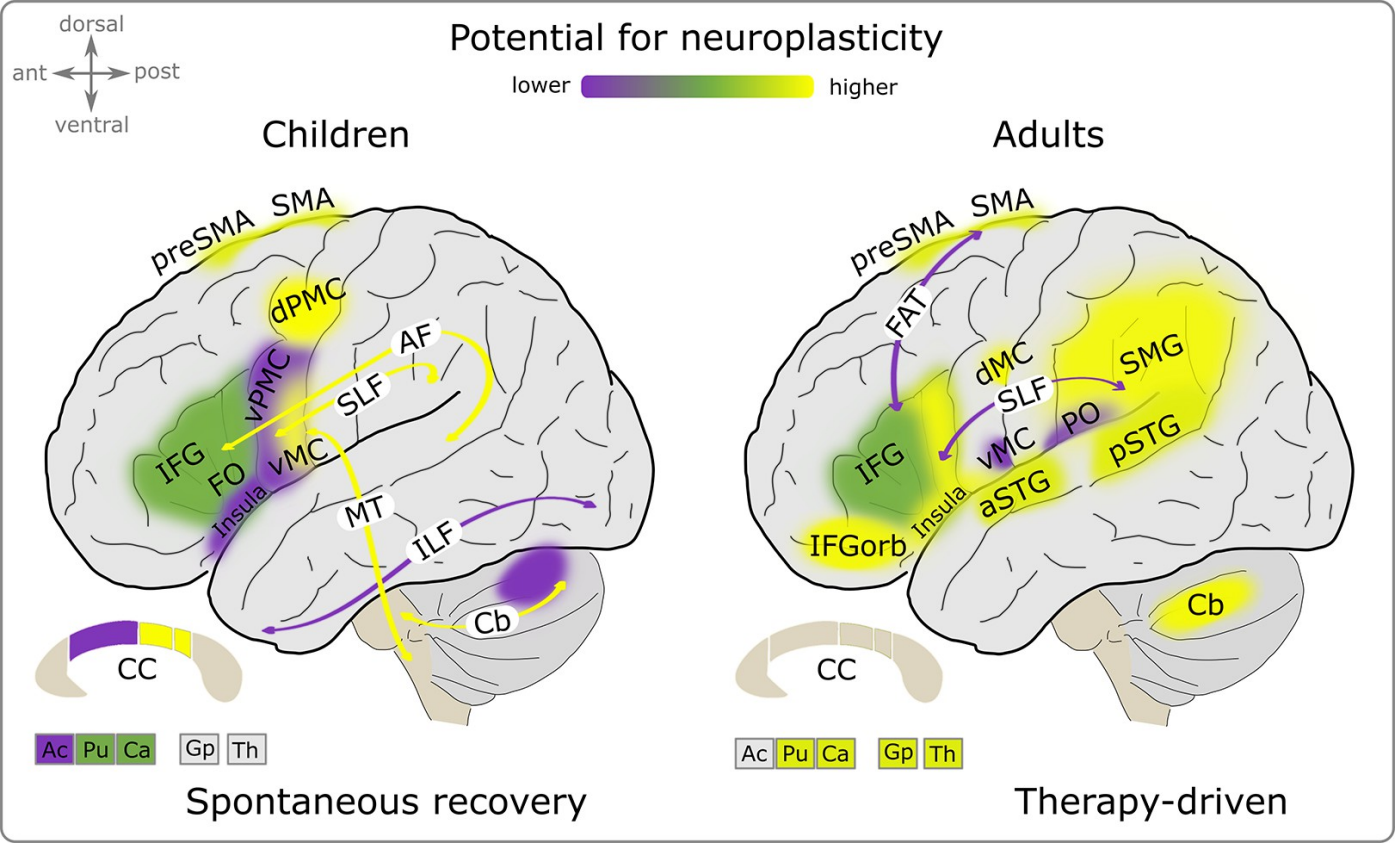
Brain regions exhibiting neuroplasticity and reorganization associated with spontaneous recovery from stuttering and therapy-induced improvements. Colored areas display key cortical brain structures and arrows illustrate fiber connections involved in stuttering and its remission. Notably, spontaneous recovery in children shows a subcortical-to-cortical structural neuroplasticity gradient [76,77,79], whereas therapy-driven improvement in adults reveals functional reorganization within and beyond the speech network [40,45,48,95–103]. Yellow areas indicate greater reorganization potential, while green areas indicate medium potential, and purple areas indicate lesser potential. The purple areas in the panel for children indicate brain structures where the volume reduction was negatively correlated with the severity of stuttering in children with persistent stuttering, while the growth rate did not change or even reversed in children with spontaneous recovery [77]. Noticeably, this is a simplified illustration; reorganization potential dependents on various factors and evidence levels vary for the individual brain regions. Abbreviations: Ac, nucleus accumbens; AF, arcuate fasciculus; aSTG, anterior superior temporal gyrus; Ca, caudate nucleus; Cb, cerebellum; CC, corpus callosum; dMC, dorsal primary motor cortex; dPMC, dorsal premotor cortex; FAT, frontal aslant tract, FO, frontal operculum; Gp, globus pallidus; IFG, inferior frontal gyrus; ILF, inferior longitudinal fasciculus; IFGorb, inferior frontal gyrus pars orbitalis; MT, motor tracts; pSTG, posterior superior temporal gyrus; PO, parietal operculum; Pu, putamen; SLF, superior longitudinal fasciculus; SMA, supplementary motor area, SMG, supramarginal gyrus; Th, thalamus; vMC, ventral primary motor cortex; vPMC, ventral premotor cortex.

The development of fluent speech involves learning to produce long motor sequences [65], which leads to the specialization of neural circuits that enable the effortless and fluid execution of fast and precise sequential movements [94]. Neuroimaging data, although scarce, has shown that initially, recovering children share neuroanatomical deficits with children with persistent stuttering, but these tend to normalize over time, with growth rates of white matter microstructure sometimes exceeding those observed in children who do not stutter [77,79]. Spontaneous recovery is primarily linked to age-related growth in white matter structures [77,79] that enable fast and accurate sequential speech movements. These white matter structures, including the corticospinal tract, superior longitudinal fasciculus, arcuate fasciculus, the somatomotor part of the corpus callosum, and cerebellar peduncles [77] (Fig 2, left panel), interconnect gray matter regions that showed significant reductions in volume in children with persistent stuttering, including the left ventral motor cortex and the left dorsal premotor cortex [76]. Recovery might require a better interareal connectivity within the speech network, a level of brain development that children with stuttering persistence do not achieve.

In addition to neuroplasticity in subcortical white matter structures, spontaneous recovery was linked with left ventral premotor cortex volume measures that were intermediate between those of children who do not stutter (controls) and children who stutter persistently [77], and with less gyrification in premotor medial areas with age, including in the presupplementary motor area and the supplementary motor area [76]. Animal studies suggest that synapses between the premotor and motor cortex in particular might support the learning of automatized chunked motor sequence output [60,104]. Spontaneous recovery could be based on a higher capacity of the ventral premotor cortex, which appears to be a crucial hub for the acquisition of speech motor skills [105,106]. The presupplementary motor area and the supplementary motor area process the metrical structure of the speech motor plan and its initiation [107], and less gyrification may indicate greater long-range connectivity of these regions during recovery, since sequential encoding, especially of long sequences, is not uniquely processed in the supplementary motor area, but is rather widespread throughout the cortical motor hierarchy [94].

Motor cortical dynamics are heavily modulated by the basal ganglia, a central subcortical hub where multiple motor pathways converge [108]. And in particular the putamen, an input zone to the motor arms of the basal ganglia [109], was characterized by a gray matter growth deficit in individuals with persistent stuttering at 3 to 5 years of age [77]. This deficit subsided with age, whereas older children (aged 6 to 12 years) with persistent stuttering began to show a gray matter deficit in the thalamus [77]. During motor skill learning and execution, motor cortical and thalamic inputs to the putamen have different functions. Animal studies suggest that corticostriatal projections are critical for motor skill learning while thalamostriatal projections are critical for the execution of learned skills [110]. It is tempting to speculate that the early gray matter deficit in the putamen is related to a deficit in learning to pronounce long speech motor sequences, while the later gray matter deficit in the thalamus relates to insufficient maturation of the subcortical motor circuits that support automated execution of such long sequences. This reasoning finds support in the observation that the cases with spontaneous recovery showed typical gray matter signal in the putamen as well as the thalamus [77].

The summarized results above align with a neurocomputational model of speech sequence learning [111], suggesting ventral premotor cortex-to-ventral motor cortex connections, first, to establish speech motor sequences via basal ganglia loops, and, second, to crystalize chunked sequences via subcortical loops through the cerebellum and corticocortical connections with the presupplementary/supplementary motor area [65]. The model suggests that early in development (prior to 2 to 3 years, typically before stuttering onset), initiation of phonemes requires a relatively high-level cortical input from the presupplementary motor area to sequentially activate the proper initiation map nodes. The basal ganglia motor loop is proposed to take over the load of sequencing through the individual phonemes in the word after a period of repeated practice, thus making production more “automatic” and freeing up higher-level cortical areas such as the presupplementary motor area [65]. According to this view, stuttering can be interpreted as an impairment of the cortico–basal ganglia loop’s role in initiation and sequencing of learned speech sequences. It is interesting that in the context of this model, the earliest occurring neural structural difference for persistent stuttering in children was in the striatum and white matter, associated with tracts that interconnect it with multiple cortical areas including premotor regions [77]. Persistent stuttering was also associated with later occurring differences in the thalamus and cerebellum. Recovery was linked to normalization of these white matter areas and greater involvement of the cerebellum. Notably, dedicated speech motor learning and automatization circuits, in particular, are implicated in stuttering recovery and persistency.

To date, longitudinal studies that have collected neuroimaging data from children who stutter are rare, and even less common are those that have examined children who recover, from the time that they are stuttering to when they eventually recover from stuttering. The limited data reported to date nevertheless facilitate emerging questions. Why does stuttering resolve in some children but not in others? What cellular and molecular mechanisms enhance or restrict the brain’s ability to develop and automate speech motor skills? Are there factors that can extend critical periods for speech motor learning? And what circumstances trigger chunking and automatization of sequences?

### Can stuttering therapy in adulthood elicit neural reorganization?

Stuttering therapy in adults can help speakers control stuttering, gain fluency, and facilitate quality of life, but a complete cure is rare to impossible, and relapse after a period of fluency is common. Interventional studies, although few, and often small in number, sample size and statistical power, have revealed patterns of potential functional reorganization within and beyond the speech network (Fig 2, right panel) that may inform future treatment strategies. Here, we differentiate 4 potential ways in which the brain may reorganize in response to behavioral intervention.

First, brain structures that are implicated in stuttering can be mobilized, indicating a neural response to the intervention. For example, fluency training increases cerebellar activity linked to learning new speech patterns [45]. Metronome-paced speech, coupled with transcranial electrical stimulation, can enhance activity in multiple brain areas that are associated with fluent speech, including the inferior frontal cortex (pars opercularis and orbitalis), anterior insula, anterior superior temporal gyrus, anterior cingulate cortex, and supplementary motor area. Subcortically, activation increases in the caudate nuclei and putamen bilaterally, and in the right globus pallidus and thalamus [95]. Brain areas (cortical and subcortical motor and auditory regions) that are less active during solo speech in stutterers were more active after 8-week practice of metronome-timed speech [96]. Finally, treatment with risperidone, a dopamine receptor 2/serotonin receptor 2 antagonist, enhanced crucial brain connections for planning speech movements, namely the left putamen, caudate nucleus, and left inferior frontal cortex [97]. These treatments target the brain structures essential for smoother speech in stuttering. In this sense, the fluency-inducing interventions can “mobilize” brain activity towards fluency in speech.

Second, brain activity and connections can be normalized. Fluency-shaping, involving slow speech, gentle vocalizations, and lighter movements, can even out brain activity differences between people who stutter and those who do not. For example, excess activity in the right frontal and parietal brain areas decreased, while reduced activity in others increased to match non-stutterers [98–100]. Additionally, connections between speech-related brain regions can become more balanced [40]. This research highlights how therapy can lead to more typical brain functioning during speech.

Third, functionally maladaptive structures can become uncoupled, suggesting the adult brain’s ability to discard ineffective pathways. Specifically, after training, a hyperactive region of the midline cerebellum showed decreased connections during rest, pointing to the brain’s adaptive mechanisms in therapy-driven stuttering improvement [101].

Finally, intact speech motor learning related structures can become more strongly integrated, underscoring the adult brain’s capacity to utilize functional connections. In other words, functional speech areas that support fluent speech that may already have established connections before therapy can become more efficient in communicating among its component structures following therapy. After fluency-shaping treatment, this stronger interaction was noticed between the left inferior frontal gyrus and the left dorsal laryngeal motor cortex, as well as between the left inferior frontal gyrus and the posterior superior temporal gyrus [102]. Essentially, practicing novel speech patterns strengthened pathways that support the integration of spectro-temporal features of speech (inferior frontal gyrus to posterior superior temporal gyrus) together with pathways that support learning to implement unfamiliar patterns of prosody production and voicing (inferior frontal gyrus to dorsal laryngeal motor cortex).

As with children’s brain development, imaging is rarely used to accompany behavioral, pharmaceutical, or noninvasive brain stimulation therapies in adults who stutter. The current data, though limited, still raise pressing questions. For example, none of the studies with adults reported morphological brain changes associated with stuttering therapy, either in gray or in white matter structures. However, while neuroplasticity patterns in children mainly relate to morphological changes (activity changes have not been studied yet), neuroplasticity patterns in adults are limited to changes in brain activity. This leads us to wonder, can stuttering therapy boost neuroplasticity in critical structures to promote recovery in children? Moreover, could therapy techniques that have been used for adults (e.g., brain stimulation) be refined and elegantly combined to facilitate neuroplasticity that supports automatic, effortless fluent speech that is characteristic of spontaneous recovery?

### What are major unsolved mysteries?

We ended each of the above sections with emerging questions. Here, we further highlight outstanding questions around 3 mysteries. Why does stuttering happen when talking but not when singing? Why does stuttering occur during communicative contexts, but not in non-communicative speech? And why do girls more often recover from stuttering than boys? Investigating the brain’s biology behind these phenomena could uncover fundamental characteristics of stuttering and reveal reasons why it persists in some individuals.

### The singing advantage

Singing dramatically eliminates instances of stuttering, even in the severest cases [112,113]. During singing, relative to speaking, the temporal structure and the coordination of laryngeal and oral movements are altered (i.e., the proportion of short phonation intervals is reduced, vowel durations are lengthened [114,115], articulation rate is slowed [115,116], and articulatory voicing is stabilized [116] in individuals who stutter). Clues to why singing enhances fluency may come from an updated understanding of the neural control of the larynx. Motor control circuits for speaking and singing largely overlap [117–119], but ventral and dorsal premotor cortex/motor cortex circuits appear to be partially involved in different laryngeal functions [120]. Only the dorsal laryngeal motor cortex (LMC) is selectively engaged in the regulation of pitch (i.e., tone and melody of song and speech [121,122]; see Box 2), while both the dorsal and ventral LMCs encode articulatory voicing (for example, the laryngeal contribution to the production of voiced and voiceless consonants [121,123]). Accordingly, different computational demands of singing and speaking might tax the LMC networks’ abilities in different ways and to different degrees (Box 2), so that the underlying neural dynamics could most likely also differ.

**Box 2. Voice in song and speech**

Both song and speech have characteristic melodies. Melodies are formed by modulating the pitch. Unlike melody in song, which is rather fixed (otherwise we would not recognize the melody), speech melody varies depending on the communicative context. Consider for example the words “Amazing grace, how sweet the sound.” When sung, the melody will always follow the same melodic pattern, but when spoken, very different pitch patterns can be employed to indicate, for example, excitement and pleasure by using a rising tone or irony by using a falling tone. It is not only the melody that is more fixed in singing compared to speech. Rhythm and volume dynamics also have a fixed temporal frame. Specifically, the temporal template in singing (i.e., the alignment of pitch, duration, and intensity) is precisely determined to correspond to a certain melody and rhythm, whereas in speaking, such temporal constraints are less definite or can be planned and executed more freely.

What do such contextual differences imply in terms of neural control? Despite the fact that there is considerable overlap between brain areas activated by song production and those activated by speech production [118,119], producing the melody of a song may more heavily involve auditory memory and feedback control mechanisms than speaking [117] to achieve the target auditory goal. By contrast, producing the melody in speech involve more degrees of freedom in feedforward control and a higher degree of automation than in singing [117]. Given the varying demands on the underlying neural network, it is plausible that the associated neurophysiological activity varies for singing versus speech production [117,119].

There are several muscle actions involved in raising pitch, lowering pitch, and articulatory voicing. Neural control coordinates the simultaneous adjustment of these muscles to bring about the desired changes in the air pressure, state of the vocal folds, the height of the larynx, and consequently, the resultant fundamental frequency [124]. Neuroimaging and neurophysiological studies have identified 2 spatially separated regions in the orofacial motor cortex with activity correlated with laryngeal movements [121,123,125–127]. Direct electrical stimulation of regions in the dorsal LMC resulted in pitch modulation during word pronunciations [122], whereas articulatory voicing was mapped to separate regions in both dorsal and ventral LMCs by direct stimulation and imaging studies [121,125].

The pitch-controlling dorsal LMC is also implied in ongoing auditory feedback control during singing, as shown by studies that compare the brain activities of amateur versus professional singers when auditory feedback was perturbed by noise-masking [128] or pitch-shifting [118]. While speaking might demand less feedback control, sentence reading under delayed auditory feedback also engages the dorsal precentral gyrus (including the dorsal LMC), but not the ventral precentral gyrus [129]. In general, the dorsal LMC is involved in pitch regulation and auditory error signal processing in singing and speaking, while no such role has been found for the ventral LMC.

In sum, pitch control in song and speech differs in its demands of feedback and feedforward control mechanisms. The dorsal LMC contains neural populations that are dedicated to encoding pitch. Its activity is enhanced under heightened demands on auditory feedback control mechanisms in speech and amateur singing, while its role diminishes with automation. Song and speech differ in their degree of automation, utilization of cognitive control, reliance on auditory memory retrieval, and the extent of affective state influence. As a consequence, song and speech will induce different activation and coupling within the brain networks involving the dorsal and ventral LMCs.

The dorsal LMC supported treatment-related improvement in speech fluency in adults who stutter who participated in a fluency-shaping program [102]. The key aspect of laryngeal control in this therapy approach is that participants learn to speak with different pitch modulation [48], voicing, volume, and timing patterns [130]. At the same time, training increases the functional coupling between the left dorsal LMC and the left inferior frontal gyrus within the sensorimotor network. Since the connectivity of the functional sensorimotor network before treatment was comparable in non-stutterers and stutterers, it can be assumed that dorsal LMC function is not altered and that the enhanced dorsal LMC connectivity after treatment indicates a compensatory role [102]. This assumption is further supported by the observation that the structural network connectivity of the dorsal LMC is intact in the same cohort of individuals who stutter [103], including rich connections with the parietal cortices allowing for intricate sensorimotor coordination and modulation of voice during speech production [131]. Against this background, the phenomenon that individuals who stutter can sing without involuntary interruptions and achieve better fluency when they control phonation during fluency shaping suggests a dedicated function of the dorsal LMC in achieving fluency. Remarkably, children who recover from stuttering exhibit an increased gray matter growth rate in the dorsal premotor cortex, a region in close proximity to the dorsal LMC [76], which is involved in auditory error signal processing to maintain fluency [129] (Box 2).

In stark contrast, individuals who stutter have weakened structural connectivity of the ventral LMC [103]; a finding reminiscent of a long-standing and robust structural abnormality in stuttering [53,132,133]. Remarkably, connectivity of the ventral LMC correlates with stuttering severity, particularly for the somatosensory cortices, inferior parietal regions, putamen, caudate nucleus, and left inferior frontal gyrus pars opercularis [103], and longitudinal data from children who continue to stutter shows reduced cortical thickness in the ventral motor cortex where the ventral LMC is located [76]. Despite ongoing research, there is persisting uncertainty regarding the specific functional contributions of the 2 laryngeal motor representations during voice production [125], and the distinct roles of dorsal and ventral LMCs in stuttering persistency and recovery remains to be verified experimentally.

Both LMCs are tightly interlinked with cortico–thalamo–cortical loops and corticocortical circuits [103,131]. Extended chains of such loops supposedly support communication across hierarchically organized cerebral networks [134,135]. This high degree of interconnectedness not only enables flexible dynamic adjustments in the temporal structure and coordination of laryngeal and oral movements during singing and speaking, but also enables differing demands on the complex interplay between sensory-guided, memory-guided, and automatic motor sequence execution. Against this background, it is promising to question how hierarchically organized circuits that align metric structures over short (speech sound voicing), medium (word accent), and long (phrasal prosody) time scales interact. Specifically, what circumstances stabilize or destabilize these hierarchies, or lead them to break down? And how do these evolving processing cascades interact with the initiation, execution, and termination of speech motor sequences?

### Social context drives stuttering

Besides the preserved ability to sing, a well-known phenomenon of interest is that stutterers are fluent when speech production occurs in a nonsocial context. Overt self-talk is free from stuttering [136]. By contrast, when speech is directed to a person or audience, or serves a communicative goal, stuttering is present. The severity of symptoms varies with the communicative context and time pressure. It might be worth exploring the idea that certain social contexts increase arousal, which leads to global changes in brain activity [137], affecting motor cortical activity and vocalization [138] and causing breakdowns of the already vulnerable speech motor system of persons who stutter. And indeed, studies with songbirds suggest that subtle aspects of acoustic structures of songs that have a crucial role in social communication are influenced by the combined effects of neuromodulator systems. Involved neuromodulator systems include cholinergic, dopaminergic, serotonergic, and noradrenergic signaling [138], systems that are influenced by changes in internal state and that are part of the ascending arousal system [139]. The ascending arousal system consists of connected brainstem nuclei known as the reticular formation. Those nuclei are tightly interlinked with the innate vocalization system. This limbic vocal system includes the periaqueductal gray, lateral reticular formation, and nucleus ambiguus, structures that support and convey emotional laughing, moaning, and crying. This system is assumed to engage in shaping the emotional tone of speech prosody [62] (Box 1). In contrast to innate vocalizations that are evoked by emotional states, human speech is learned and volitional. Still, central mechanisms for the interaction between limbic and cognitively controlled vocalization pathways remain largely unknown, as are their potential interactions with stuttering. Possible open questions are: how do affect, social context, and goal-directed communication shape network formations or hierarchies of speech production circuits; is there competition between the volitional articulation motor network and the innate vocal motor network; and if so, what drives such a competition, which brain structures are involved, and to what extent might this influence stuttering? Following this line of research might facilitate the rationale for cognitive behavioral therapy approaches to help control feelings such as insecurity, anxiety, self-doubt, shame, or anger.

Beyond arousal, the social context offers significant potential to shape speech. Communication is inherently human and relies on active listening and response. This mutual exchange makes communication rewarding, driving speech motor learning through reinforcement. Successful communication and listener feedback fuel the refinement of speech skills, blending physical vocal coordination with psychological rewards. The nucleus accumbens is a striatal structure that tightly interlinks motor and limbic circuits and that is involved in the coordination of cognition, emotion and action [140], and social motivation [141], but also in active and inhibitory avoidance and reward seeking [142,143]. Notably, this region in the ventral striatum is altered in children and adults who stutter. That is, children have decreased gray matter volume in the ventral striatum that scales with stuttering severity [77], while adults have enlarged substrate in the right hemisphere [46,144]. Given these contradictory findings, it holds potential promise to inquire how reward-related brain structures and speech motor coordination structures interact in varying social contexts, and how these develop with age: for example, are there critical biological or social periods throughout the lifetime where the dynamics of supposed interactions change? Future research might disentangle whether and how limbic structures, and in particular, the nucleus accumbens, are involved in the chronic manifestation of stuttering. On the other hand, one might ask whether relevant neural circuits shape the establishment of avoidance behavior that might be related to proactive action inhibition (avoidance of certain communicative situations, words, or sounds) or reactive action inhibition (the modification of stuttering events right when they occur)? In other words, are these to be understood as part of the core deficits of stuttering, or do they reflect the mere impact of experiencing this communication disorder (i.e., related feelings when communication fails or is expected to fail, including fear, frustration, and depression)?

### Sex differences

Whether a child is a boy or a girl has significant impacts on the chances of stuttering recovery: while the male-to-female ratio is approximately 1:2 at stuttering onset in early childhood, only 1 out of 5 adults with persistent stuttering is a woman [5]. Sex effects are commonly associated with neurodevelopmental disorders, with male sex being a risk factor for childhood speech and language disorders [6,8]. It is likely that sexual dimorphisms are driven by genetic, cellular, and molecular mechanisms, and sex hormones might be a pivotal starting point to approach hypotheses. Thus, for example, prepubertal girls already have higher estradiol levels then boys, while the serum-level of this sex hormone increases with age and pubertal stage in girls and boys [145], and heavily fluctuates in girls with the menstrual cycle that starts with puberty. Changes in sex hormones might influence functional connectivity, neurotransmission, and brain structure [146]. For example, estrogen, with estradiol as its primary form during reproductive years, exhibits neurotrophic and neuroprotective properties that act on the striatum, cerebral cortex, and hippocampus [147]. Estrogen receptors colocalize with neurotransmitter pathways including the glutamatergic, dopaminergic, GABA-ergic, and serotonergic systems, and seem implicated in neurite outgrowth, synaptogenesis, dendritic branching, myelination, and other neuroplasticity mechanisms [146].

Distinct actions of estrogen on neural vocal learning and vocalization pathways have been outlined in songbirds. In certain songbird species, sexual dimorphism displays with limited to no female vocal learning. Estradiol treatment in female zebra finches revealed that the sexual dimorphism in song learning ability was established by the interaction between sex hormone signaling and sex chromosome gene expression in the song system anatomy during development [148]. Of course, one might critically ask, whether such sex-related interactions translate to human speech motor learning. Initial indications come from a pilot study with infants, 4 and 8 weeks old. At 8 weeks, their cry melody properties were correlated with serum concentrations of estradiol that varied with sex [149]. Cry melodies were analyzed with respect to frequency modulation skills, and the complexity of those melodies increased with increasing serum estradiol. This association indicates that sex hormones might influence the speech motor learning system well before puberty. Against this background, it might be reasonable to ask whether estrogen levels shape the neuroplasticity potential within the speech motor system thereby pushing girls more often towards sustained fluency.

## Conclusions

Stuttering is complex and multifaceted, with numerous open questions relevant to its neurobiological bases. This Unsolved Mystery aimed to provide an overview of the evolving science of stuttering, highlighting key unanswered questions to help stimulate discussions toward further research. Though multiple topics were discussed, we note that not all promising topics relevant to neurobiology of stuttering could be covered in this limited space. For example, examining neurophysiological aspects of stuttering using electrophysiological methods have the potential to reveal temporal dynamics of the reported cortico–basal ganglia loop function in stuttering through examining neural oscillations and synchrony of selected frequency band oscillations. Moreover, neurophysiological measures can provide insights into what happens neurologically at the time when speech fluency is interrupted. Additional topic areas that warrant further investigation include, but are not limited to: how we can address variability within the stuttering population; speech motor skill acquisition versus automatization; the role of action inhibition and the right hemisphere homologues in stuttering; hyperdirect connectivity of opercular speech network to the subthalamic nucleus [150]; and inter-hemispheric integration and corpus callosum development as they relate to stuttering persistence and recovery.

Highly relevant to the topic of singing, is the role of intrinsic timing and rhythm as they influence stuttering severity and recovery, and how they relate to the rhythm deficit hypothesis [151], which have implications for possible common, core deficits that present transdiagnostically across disparate neurodevelopmental disorders affecting speech and language function. Neural network analyses leveraging multimodal imaging data and neurocomputational modeling, as well as new discoveries of stuttering gene loci [152] could help identify the presence of neural subtypes within the stuttering population; this could lead to better targeting of interventions that are based on each individual’s subtype designation. Systemic investigations into these and other questions may bring us to the cusp of breakthroughs, not only in elucidating the nature of stuttering, but also in enhanced clinical management of its core symptoms in the future.
